# Effects of supplemented isoenergetic diets varying in cereal fiber and protein content on the bile acid metabolic signature and relation to insulin resistance

**DOI:** 10.1038/s41387-018-0020-6

**Published:** 2018-03-07

**Authors:** Martin O. Weickert, John G. Hattersley, Ioannis Kyrou, Ayman M. Arafat, Natalia Rudovich, Michael Roden, Peter Nowotny, Christian von Loeffelholz, Silke Matysik, Gerd Schmitz, Andreas F. H. Pfeiffer

**Affiliations:** 1 Department of Endocrinology & Diabetes, University Hospitals Coventry and Warwickshire NHS Trust, Coventry, CV2 2DX UK; 2 Centre for Applied Biological & Exercise Sciences, Coventry University, Coventry, CV1 5FB UK; 3 Division of Translational & Experimental Medicine, Warwick Medical School, University of Warwick, Coventry, CV4 7AL UK; 4 Human Metabolic Research Unit, University Hospitals Coventry and Warwickshire NHS Trust, Coventry, UK; 5 Aston Medical Research Institute, Aston Medical School, Aston University, Birmingham, B4 7ET UK; 6 Department of Clinical Nutrition, German Institute of Human Nutrition, 14558 Potsdam-Rehbruecke, Germany; 7 Department of Endocrinology, Diabetes and Nutrition, Campus Benjamin Franklin, Charité-University-Medicine Berlin, Berlin, Germany; 8 Division of Diabetology and Endocrinology, Department of Internal Medicine, Spital Bülach, Bülach, Switzerland; 9 Division of Endocrinology and Diabetology, Medical Faculty, Heinrich-Heine University Düsseldorf, 40225 Düsseldorf, Germany; 10 German Center for Diabetes Research, München-Neuherberg, Germany; 11 Institute for Clinical Diabetology, German Diabetes Center (Leibniz Center for Diabetes Research), Heinrich-Heine University Düsseldorf, 40225 Düsseldorf, Germany; 12 Department of Anaesthesiology and Intensive Care and Integrated Research and Treatment Center, Center for Sepsis Control and Care (CSCC), Friedrich Schiller University, Jena, Germany; 13 Institute for Clinical Chemistry and Laboratory Medicine, University Hospital Regensburg, Regensburg, Germany

## Abstract

Bile acids (BA) are potent metabolic regulators influenced by diet. We studied effects of isoenergetic increases in the dietary protein and cereal-fiber contents on circulating BA and insulin resistance (IR) in overweight and obese adults. Randomized controlled nutritional intervention (18 weeks) in 72 non-diabetic participants (overweight/obese: 29/43) with at least one further metabolic risk factor. Participants were group-matched and allocated to four isoenergetic supplemented diets: control; high cereal fiber (HCF); high-protein (HP); or moderately increased cereal fiber and protein (MIX). Whole-body IR and insulin-mediated suppression of hepatic endogenous glucose production were measured using euglycaemic–hyperinsulinemic clamps with [6-6^2^H_2_] glucose infusion. Circulating BA, metabolic biomarkers, and IR were measured at 0, 6, and 18 weeks. Under isoenergetic conditions, HP-intake worsened IR in obese participants after 6 weeks (*M*-value: 3.77 ± 0.58 vs. 3.07 ± 0.44 mg/kg/min, *p* = 0.038), with partial improvement back to baseline levels after 18 weeks (3.25 ± 0.45 mg/kg/min, *p* = 0.089). No deleterious effects of HP-intake on IR were observed in overweight participants. HCF-diet improved IR in overweight participants after 6 weeks (*M*-value 4.25 ± 0.35 vs. 4.81 ± 0.31 mg/kg/min, *p* = 0.016), but did not influence IR in obese participants. Control and MIX diets did not influence IR. HP-induced, but not HCF-induced changes in IR strongly correlated with changes of BA profiles. MIX-diet significantly increased most BA at 18 weeks in obese, but not in overweight participants. BA remained unchanged in controls. Pooled BA concentrations correlated with fasting fibroblast growth factor-19 (FGF-19) plasma levels (*r* = 0.37; *p* = 0.003). Higher milk protein intake was the only significant dietary predictor for raised total and primary BA in regression analyses (total BA, *p* = 0.017; primary BA, *p* = 0.011). Combined increased intake of dietary protein and cereal fibers markedly increased serum BA concentrations in obese, but not in overweight participants. Possible mechanisms explaining this effect may include compensatory increases of the BA pool in the insulin resistant, obese state; or defective BA transport.

## Introduction

Isoenergetic changes of the dietary macronutrient composition can have significant effects on insulin resistance (IR)^1, 2^. A high intake of insoluble cereal fibers (HCF) is associated with a 20–30% reduced risk of developing type-2 diabetes (T2DM)^3, 4^. In contrast, high-protein (HP) intake in sedentary subjects, in the absence of sustained weight loss, may increase this risk by the same magnitude^5, 6^. Furthermore, HP-intake during weight loss with a hypocaloric diet appears to eliminate weight loss-induced improvement of IR in obese subjects^7^. HP-induced IR could be related to activation of the mammalian-target-of-rapamycin (mTOR) S6-kinase-1 (S6K1) signaling cascade^8–12^, with negative effects on IR possibly being prevented when co-ingesting additional cereal fibers^12^ or plant derived (also fiber-rich) sources of protein^6, 13, 14^, thereby inhibiting the digestion and/or absorption of dietary protein^12^.

However, additional mechanisms are likely to be involved and the signaling potential of bile acids (BA) in the systemic circulation could play a key role in this context^15^. Besides their established role in digestion, BA are potent regulators of metabolism^16–18^ that can be influenced by diet^17, 19–22^. BA-uptake by the ileal enterocyte is important for regulation of BA synthesis and gut-liver signaling^15^. When transiting through the ileal enterocyte, BA activate farnesoid-X-nuclear-receptor (FXR) and increase fibroblast growth-factor-19 transcription (FGF-19; mouse ortholog, FGF-15). Thereafter, FGF-19 is released from the intestine to inhibit hepatic BA synthesis^23^, and regulates aspects of glucose and lipid metabolism via mainly insulin-like actions^24–27^. BA-activation of FXR in hepatocytes increases small-heterodimer-partner (SHP) expression, leading to decreased expression of sterol-regulatory-element binding-protein (SREBP1c) and lipogenesis; and reduced expression of glucose-6-phosphatase (G6Pase) and phospoenolpyruvate-carboxykinase (PEPCK), thereby reducing gluconeogenesis. Thus, changing the levels and/or composition of circulating BA could have a role for treating IR and related conditions^28–30^.

HP-diets may regulate BA-metabolism by modulating genes involved in energy metabolism and uncoupling^17, 19, 21^, whereas HCF-rich diets influence both BA binding and BA reabsorption from the gut via their lignin/cellulose contents^20, 22, 31, 32^. Notably, diet-induced metabolic effects appear to be different between obese, more insulin-resistant and non-obese, less insulin-resistant subjects^33, 34^. To date, the effects of isoenergetic, HP and/or HCF supplemented diets on circulating BA and their relation to adiposity and changes in IR have not been investigated in controlled human interventions. To increase power, we additionally investigated potential differences between overweight and obese subjects in BA signatures and associated metabolic markers in the pooled cohort (all 4 dietary groups combined).

## Subjects and methods

### Study population

This study presents additional analyses from the Protein, Fiber and Metabolic Syndrome (ProFiMet) data set (clinicaltrials.gov, NCT00579657), described in our core publication^12^. The Ethics Committee of the University of Potsdam approved the study (BMBF-FKZ-0313826). All participants provided written informed consent. Herein, we present results of a sub-group of 72 participants from the ProFiMet study who successfully had completed the 18 weeks dietary intervention (i.e., who had attended all study days, maintained their body weight, had complete data from dietary monitoring, did not change their exercise habits and did not take drugs known to affect IR (i.e., steroids, antibiotics, or acetylic salicylic acid^35^)); and had complete measurements of circulating BA (weeks 0, 6, and 18). All participants: (i) were Caucasian; (ii) were either overweight (BMI ≥ 25 but <30 kg/m^2^; *n* = 29) or obese (BMI ≥ 30 kg/m^2^; *n* = 43); (iii) had waist circumference >80 cm in females and >94 cm in males; and (iv) had at least one more feature of the metabolic syndrome (but no diabetes) according to the International Diabetes Federation definition/criteria^36^. Participants underwent an oral glucose tolerance test prior to the intervention. Baseline characteristics in the four dietary groups were comparable (all *p* > 0.1; Table 1). Characteristics when comparing overweight and obese participants from the pooled dietary groups are presented in Table 2.

**Table 1:** Characteristics of the dietary interventions and baseline characteristics of the study subjects (*n* = 72)

Diet characteristics	Control	HCF	HP	MIX	*p*-value
Macronutrient content (% of energy)					
Carbohydrates	55	55	40–45	45–50	—
Protein	15	15	25–30	20–25	—
Fat	30	30	30	30	—
Cereal fiber	Not emphasized	Emphasized	Not emphasized	Emphasized	—
Matched characteristics					
Age (years)	54.4 ± 1.6	54.6 ± 2.9	55.7 ± 2.6	55.2 ± 1.8	0.98
Gender (females/males)	12/8	10/5	11/7	13/6	0.94
Use of drugs^a^ (*n*)	9	8	9	10	0.78
Waist circumference (cm)	101.1 ± 1.8	101.1 ± 2.0	102.7 ± 3.0	100.4 ± 3.1	0.93
BMI (kg/m^2^)	30.7 ± 0.7	31.8 ± 0.8	31.9 ± 0.9	31.3 ± 0.9	0.69
Additional characteristics					
Overweight/obese	10/10	5/10	6/12	8/11	0.70
Glucose metabolism^b^ (NGM/PGM)	11/9	11/4	6/12	11/8	0.26
*M*-value (mg/kg/min)	4.44 ± 0.42	3.79 ± 0.39	3.92 ± 0.41	4.14 ± 0.27	0.65
EGP (mg/kg/min)	1.67 ± 0.03	1.65 ± 0.04	1.59 ± 0.05	1.60 ± 0.05	0.43
HEP-IR (mg/kg/min)	16.11 ± 1.59	13.99 ± 1.35	16.51 ± 1.64	16.19 ± 2.38	0.78
HOMA-IR	2.13 ± 0.21	1.79 ± 0.21	2.15 ± 0.16	2.16 ± 0.41	0.77
Body fat mass (kg)	33.79 ± 1.49	37.92 ± 2.02	36.64 ± 2.42	36.81 ± 2.93	0.61
Body lean mass (kg)	53.24 ± 2.82	55.10 ± 2.85	54.73 ± 2.79	51.80 ± 2.32	0.82
Liver fat (%)	8.66 ± 2.29	7.75 ± 2.11	6.34 ± 1.54	9.65 ± 2.63	0.74
VAT (L)	4.39 ± 0.38	3.88 ± 0.47	4.87 ± 0.44	4.58 ± 0.57	0.54
NVAT (L)	15.13 ± 0.73	16.32 ± 0.95	16.94 ± 1.49	16.57 ± 1.79	0.75
REE (kcal/d)	1476 ± 49	1575 ± 63	1547 ± 76	1412 ± 60	0.27
Pooled BA (μmol/L)	1.81 ± 0.33	2.28 ± 0.68	1.84 ± 0.43	1.36 ± 0.23	0.52
Primary BA (μmol/L)	1.08 ± 0.27	1.09 ± 0.27	0.96 ± 0.28	0.74 ± 0.17	0.71
Secondary BA (μmol/L)	0.53 ± 0.06	0.54 ± 0.10	0.63 ± 0.12	0.48 ± 0.07	0.70
Tertiary BA (μmol/L)	0.12 ± 0.02	0.40 ± 0.24	0.15 ± 0.03	0.09 ± 0.01	0.15
FGF-19 (pg/mL)	124.3 ± 21.1	111.6 ± 12.2	104.8 ± 13.8	106.5 ± 15.5	0.83

Participants were randomly allocated to one of four isoenergetic diets (i.e., control, high cereal fiber (HCF), high protein (HP), or moderately high in both cereal fiber and protein (MIX)) using a computerized group-matching algorithm ensuring homogeneity of the main variables, (matched characteristics by randomization: age, sex, waist circumference, body mass index (BMI), and drug intake)

Data presented as mean of absolute values ± standard error for continuous variables, and as absolute numbers and proportions for categorical variables. Comparison between dietary groups was performed using one-way ANOVA, with diet as the discriminating factor

*HCF* diet high in cereal fiber, *HP* diet high in protein, *MIX* diet moderately high in both cereal fiber and protein, *BMI* body mass index, *NGM* normal glucose metabolism based on oGTT, *PGM* pathological glucose metabolism based on oGTT showing impaired fasting glucose and/or impaired glucose tolerance, *M-value* insulin-mediated glucose uptake as a measurement of whole-body insulin sensitivity, *EGP* endogenous glucose production, *HEP-IR* hepatic insulin resistance, *HOMA-IR* homeostasis model assessment for insulin resistance, *VAT* visceral adipose tissue, *NVAT* non-visceral adipose tissue, *REE* resting energy expenditure, *BA* bile acids, *FGF-19* fibroblast growth factor-19

^a^Use of lipid-lowering and/or antihypertensive drugs

^b^Data derived from an oral glucose tolerance test (oGTT)

**Table 2:** Baseline characteristics (week 0) of the study participants from the four dietary intervention groups when pooled and categorized as overweight (BMI ≥ 25 and <30 kg/m^2^; *n* = 29) or obese (BMI ≥ 30 kg/m^2^; *n* = 43)

Participant characteristics	Overweight (*n* = 29)	Obese (*n* = 43)	*p*-value
Age (years)	54.6 ± 1.5	55.6 ± 1.6	NS
Sex (female/male)	18/11	28/15	NS
Lipid lowering and/or antihypertensive drugs	16	20	NS
Body weight/composition			
Weight (kg)	81.3 ± 2.0	95.6 ± 2.0	<0.005
Height (m)	1.7 ± 0.02	1.7 ± 0.01	NS
BMI (kg/m^2^)	28.2 ± 0.2	33.5 ± 0.4	<0.005
Waist circumference (cm)	95.1 ± 1.5	105.8 ± 1.6	<0.005
Fat mass (kg)	29.4 ± 0.8	40.7 ± 1.4	<0.005
Lean mass (kg)	51.8 ± 2.1	54.8 ± 1.7	NS
VAT (l)	3.6 ± 0.3	4.9 ± 0.3	<0.005
SCAT_a_ (l)	22.6 ± 0.4	26.4 ± 0.5	<0.005
Liver fat (IHL) (%)	4.5 ± 0.7	10.5 ± 1.7	0.001
Insulin sensitivity			
*M*-value (mg/kg/min)	4.5 ± 0.2	3.8 ± 0.3	0.03
EGP (mg/kg/min)	1.7 ± 0.03	1.6 ± 0.03	0.008
HEP-IR (mg/kg/min)	13.7 ± 1.1	17.1 ± 1.3	0.04
HOMA-IR	1.7 ± 0.1	2.3 ± 0.1	0.006
Glucose metabolism (oGTT)			
NGM/PGM	19/10	18/25	NS
Blood pressure (mmHg)			
Systolic	138.3 ± 2.6	143.0 ± 3.2	NS
Diastolic	88.4 ± 2.0	94.6 ± 2.0	NS
RQ	0.8 ± 0.01	0.7 ± 0.01	NS
REE (kcal/day)	1415 ± 51	1556 ± 38	NS
Cholesterol			
Total (mmol/L)	5.1 ± 0.1	5.4 ± 0.1	NS
HDL (mmol/L)	1.4 ± 0.06	1.3 ± 0.04	NS
LDL (mmol/L)	3.3 ± 0.1	3.5 ± 0.1	NS
Triglycerides (mmol/L)	1.0 ± 0.08	1.34 ± 0.1	0.007
Free fatty acids (mmol/L)	0.6 ± 0.04	0.7 ± 0.04	NS
HbA1c (%)	5.0 ± 0.06	5.2 ± 0.05	NS
Biomarkers of protein intake			
Urine nitrogen/creatinine ratio	7.31 ± 0.4	7.95 ± 0.4	NS
Fecal isovalerate (mmol/L)	3.2 ± 0.3	3.0 ± 0.3	NS
FGF-19 (pg/mL)	105.54 ± 9.21	116.55 ± 12.28	NS

Data are presented as means ± standard error for continuous variables, and as absolute numbers and proportions for categorical variables. Comparison between overweight and obese participants was performed using one-way ANOVA.

*BMI* body mass index, *VAT* visceral adipose tissue, *SCATa* abdominal subcutaneous adipose tissue, *IHL* intrahepatic fat content, *M*-value insulin-mediated glucose uptake as a measurement of whole-body insulin sensitivity, *EGP* endogenous glucose production, *HEP-IR* hepatic insulin resistance, *HOMA-IR* homeostasis model assessment for insulin resistance, *oGTT* oral glucose tolerance test, *NGM* normal glucose metabolism based on oGTT, *PGM* pathological glucose metabolism based on oGTT showing impaired fasting glucose and/or impaired glucose tolerance, *RQ* respiratory quotient, *REE* resting energy expenditure, *HDL* high density lipoprotein, *LDL* low density lipoprotein, HbA1c hemoglobin A1c, *FGF-19* fibroblast growth factor-19

### Dietary intervention

Details of this dietary intervention have been published^12^. In brief, participants were group-matched according to age, sex, waist circumference, BMI and drug intake and randomly allocated to one of four moderately fat reduced (30% of energy content) diets, varying in protein and cereal fiber content (control (C); high cereal fiber (HCF); high protein (HP); or moderately increased cereal fiber and protein (MIX)). (Table 1). All diets were isoenergetic and based on assumed healthy foods (details are provided in supplementary materials, including Supplementary Table 1). In the HP-group, high-fat animal-protein sources were restricted. Participants in all 4 dietary groups received tailored supplements (4 lots, containing 63 portions each at weeks 0, 3, 6, and 12, respectively) for twice daily consumption throughout the 18 weeks intervention. A selection of drinking powders in five different flavors, together with a purpose-made shaker; and a baking mix for the preparation of pancakes was provided in each dietary group. Supplements were dissolved in low fat (1.5%) milk, 200 mL per portion when using drinking powders; or 120 mL when using baking mixes, as per the participant’s preference but ideally in a 1:1 ratio. Supplements provided to the control group were based on a low-fiber grain mixture, which also served as carrier for the cereal fiber and protein-enriched supplements in the other intervention groups. The HCF dietary supplements were enriched with 2 × 15-g insoluble cereal fiber extracts from oat hulls; the HP dietary supplements contained 2 × 29-g isolates from whey and pea proteins, and the supplements provided to the mix groups contained 2 × 8-g cereal fiber extracts from oat hulls and 2 × 19-g protein isolates from whey and peas. During the first 6 weeks, participants weighed all foods and provided detailed information on processing, cooking, and brand names. According to daily food protocols during the first 6 weeks, the achieved percent intakes of protein and carbohydrates and intake of cereal fiber per day were 17%, 52%, and 14 g in controls; 17%, 52%, and 43 g in HCF; 28%, 43%, and 13 g in HP; and 23%, 44%, and 26 g in MIX. Differences in protein intake between groups were balanced by modulating carbohydrate intake. Fat contents were comparable between diets (total fat intake (g), *p* > 0.45; percent fat intake, *p* > 0.14 at all study days). Biomarkers for protein intake (urine ratio nitrogen/creatinine; fecal isovaleric acid) and fermentable fiber intake (fecal butyrate; breath hydrogen) were used to monitor dietary adherence^12^.

### Body composition and liver fat content

Anthropometric measurements were performed by trained staff, using standard methods. Body fat content was assessed using 1.5T magnetic resonance imaging (Magnetom Avanto, Siemens Healthcare, Erlangen, Germany)^12^. Proton magnetic resonance spectroscopy (^1^H-MRS) was utilized to assess hepatic lipid content^12, 37^.

### Whole-body insulin resistance and insulin-induced suppression of hepatic glucose production

Euglycemic–hyperinsulinemic clamps (40 mU/kg/min) were performed in the overnight fasted state at weeks 0, 6, and 18 (*n* = 15–20 participants/group and study day) to assess whole-body IR (expressed as *M*-value). Tracer experiments were performed in a matched subset of participants (*n* = 9–11 participants/group and study day), to measure endogenous (hepatic) glucose production (EGP) and EGP-suppression by insulin infusion. Hepatic IR (HEP-IR) was estimated as EGP x fasting plasma insulin (FPI)^37^.

### Sample preparation

Blood samples were drawn in timed intervals at baseline and throughout the clamp experiments, following 12-h overnight fasting. Samples were immediately chilled and centrifuged, and the supernatant fluid was aliquoted and stored at −80 °C until analyzed.

### Bile acid (BA) measurement

Plasma BA were analyzed using liquid chromatography-tandem mass spectrometry (LC–MS/MS), as described^38^. The LC–MS/MS system consisted of an API-4000-QTrap (AB-Sciex, Darmstadt, Germany) coupled with electrospray ionization interface operated in the multiple-reaction monitoring positive ion-mode. Chromatographic separation was performed on Agilent-1200 HPLC system (Agilent, Waldbronn, Germany) with a Kinetex C18 column (50 × 2.1 mm, 2.6 µm; Phenomenex). Details are provided in the supplementary materials. The categorization of key BA is summarized in Supplementary Table 2.

### Measurement of butyric acid in fecal samples

Assessment of fecal butyric acid deficiency was used as an indirect indicator of impaired intestinal barrier function^39^. Details are provided in the supplementary materials.

### Other biomarkers

FGF-19 was measured by enzyme-linked immunosorbent assay (R&D Systems, Minneapolis, MN). All other biomarkers in blood and hydrogen breath tests were measured as detailed previously^12^.

### Study sample size justification

Power calculation for the detection of diet-induced changes in IR was initially based on 26 participants for each dietary group and an assumed dropout rate of 30% per group^12^. Significant diet-induced changes in IR were also observed in the herein presented sub-cohort of participants with available complete data on BA measurements. Further, a recent study by Haeusler et al.^40^ showed increased BA synthesis in human obesity, with a sample size of 32 obese and 11 non-obese non-diabetic Caucasian adults. Therefore, a sample size of 43 obese and 29 non-obese participants in our study was assumed to be sufficient.

### Statistical analyses

Data are presented as means ± standard error (SEM). Significance was defined as *p* < 0.05. Analysis of variance (ANOVA) and *t*-tests were used to compare study subgroups. Two-way ANOVA was additionally used, with factors of obesity and dietary group, to investigate changes in BA concentrations. Repeated measures ANOVA, with dietary group as the factor, was used for longitudinal comparison between dietary groups and time, with corrections for sphericity applied. Log-transforms were applied for not normally distributed data. Forward stepwise regression, Pearson’s correlation and partial correlations (adjusted for BMI and age^41^, with gender showing no influence in our analyses) were used to test relationships between BA concentrations and measures of IR. Analyses were performed using SPSS version 24 (SPSS Inc., Chicago, IL).

## Results

### Diet-induced changes on insulin resistance

At baseline, IR was comparable between dietary groups (Table 1). Both whole-body IR (*M*-value) and EGP differed between dietary groups at 6 weeks (*M*-value, *p* = 0.048; EGP, *p* = 0.005), driven by opposite changes in the HP and HCF groups: *M*-value improved in the HCF group at 6 weeks (3.80 ± 0.39 (week-0) vs. 4.15 ± 0.34 (week-6) mg/kg/min; *p* = 0.037), but was not different from baseline at 18 weeks (*p* = 0.11). In the HP group, *M*-value worsened after 6 weeks (3.93 ± 0.41 (week-0) vs. 3.31 ± 0.32 (week-6) mg/kg/min; *p* = 0.011), but was not different from baseline after 18 weeks (*p* = 0.46). *M*-value did not change in control and MIX, neither after 6 nor 18 weeks. Estimated hepatic IR (expressed as HEP-IR = EGP × FPI)^37^ was unaffected by the dietary intervention (*p* > 0.65).

### Diet-induced changes in IR when comparing overweight vs. obese participants

At baseline, IR was significantly different between overweight and obese individuals (Table 2), as expected. When consuming HCF for 6 weeks, whole-body IR improved in overweight (*M*-value: 4.25 ± 0.35 (week-0) vs. 4.81 ± 0.31 (week-6) mg/kg/min; *p* = 0.016), but not in obese participants (*p* = 0.29). In contrast, HP-diet for 6 weeks worsened IR in obese (*M*-value: 3.77 ± 0.58 (week-0) vs. 3.07 ± 0.44 (week-6) mg/kg/min; *p* = 0.038), but not in overweight participants (*p* = 0.18). Control and MIX diets did not influence IR, neither in obese nor in overweight participants (all *p* > 0.42).

After 18 weeks, improved IR in overweight participants in the HCF group (*M*-value: 4.25 ± 0.35 (week-0) vs. 5.12 ± 0.47 (week-18) mg/kg/min; *p* = 0.055) and worsened IR in obese participants in the HP-group (*M*-value: 3.77 ± 0.58 (week-0) vs. 3.25 ± 0.45 (week-18) mg/kg/min; *p* = 0.089) tended to be maintained, but statistical significance was not reached. Control and MIX diets did not influence IR, neither in obese nor in overweight participants (all *p* > 0.29).

### Dietary factors influencing BA-circulating profiles

**Fig. 1:**
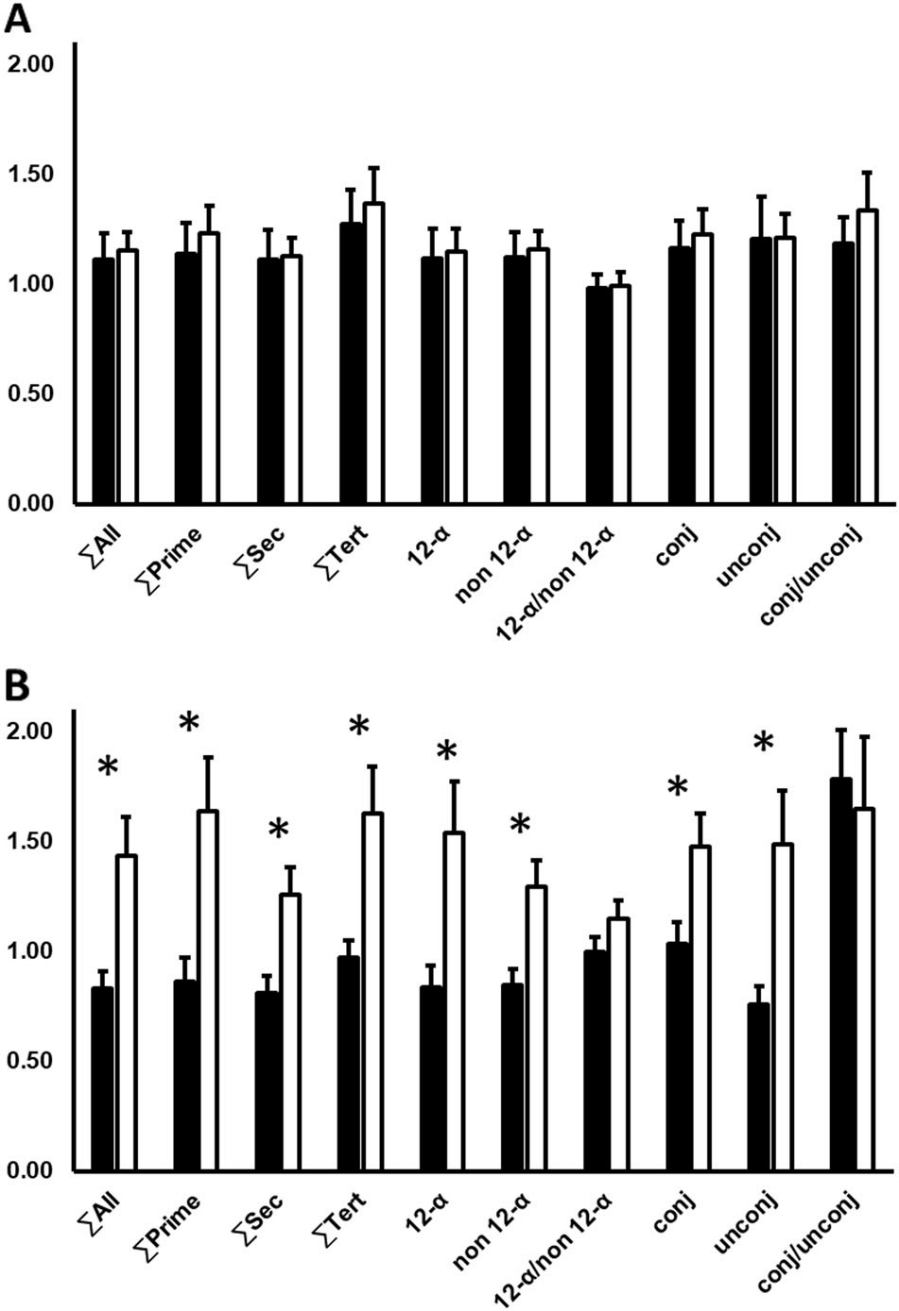
Comparison of bile acids (BA) circulating concentrations between obese (*n* = 43; white bars) and overweight (*n* = 29; black bars) participants in the pooled cohort, irrespective of the dietary groups. The graph shows changes relative to the baseline values (week 0) after **a** 6 weeks and **b** 18 weeks of dietary intervention. *Statistically significant at alpha < 0.05. ∑All: sum of all BA; ∑Prim: sum of primary BA; ∑Sec: sum of secondary BA; ∑Tert: sum of tertiary BA; 12-α: 12-α hydroxyl BA; non 12-α: non 12-α hydroxylated BA; 12-α/non 12-α: ratio of 12-α hydroxyl BA to non 12-α hydroxylated BA; Conj: conjugated BA; Unconj: unconjugated BA; Conj/Unconj: ratio of conjugated to unconjugated BA

**Fig. 2:**
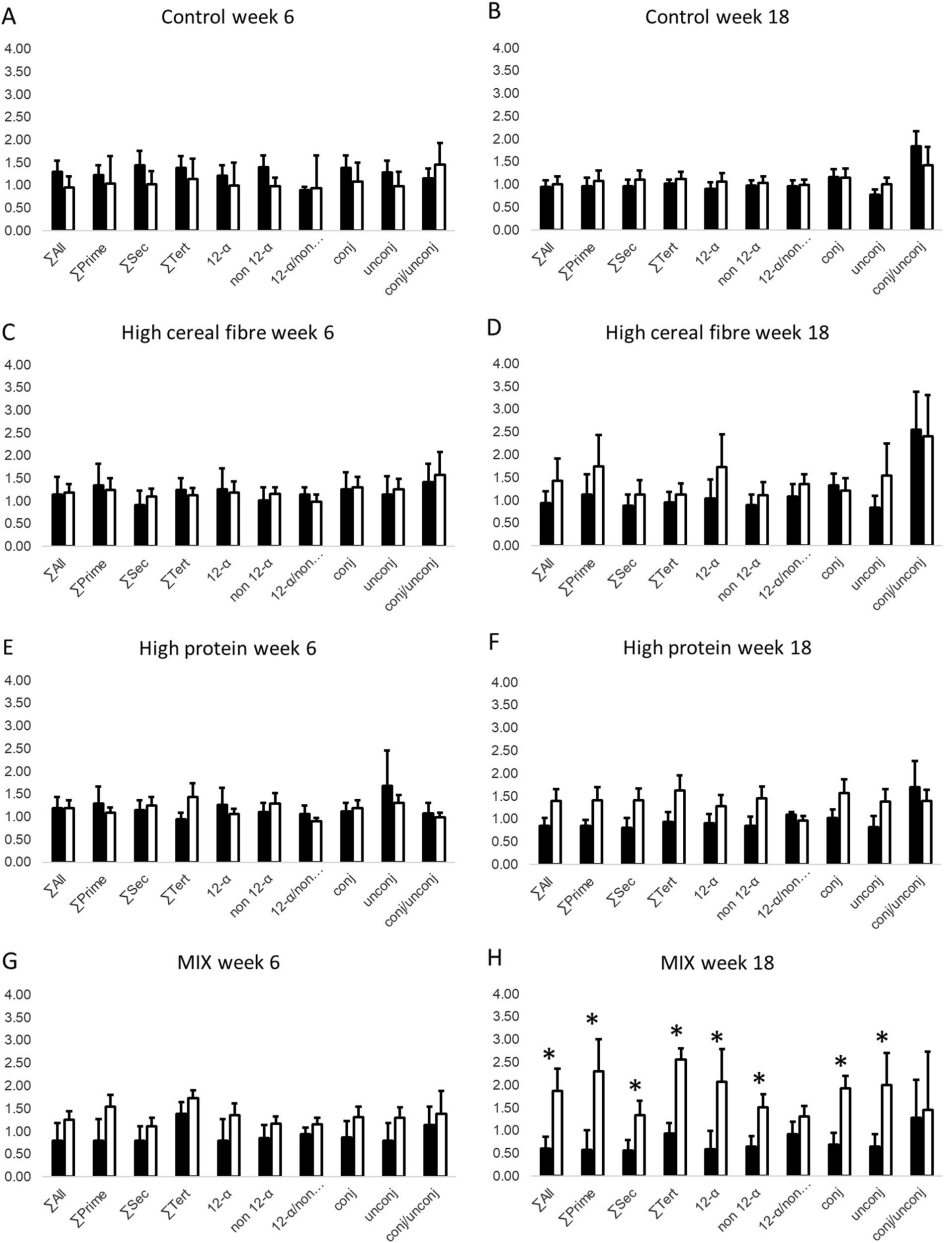
Comparison of circulating bile acids (BA) concentrations, relative to baseline value at week 0, after 6 and 18 weeks between obese (ob) and overweight (ow) subjects in the 4 diet groups : **a** Control at 6 weeks (ow/ob: *n* = 10/10). **b** Control at 18 weeks (ow/ob: *n* = 10/10). **c** High cereal fiber (HCF) at 6 weeks (ow/ob: *n* = 5/10). **d** HCF at 18 weeks (ow/ob: *n* = 5/10). **e** High protein (HP) at 6 weeks (ow/ob: *n* = 6/12); **f** HP at 18 weeks (ow/ob: *n* = 6/12). **g** Mixed-diet (MIX) group at 6 weeks (ow/ob: *n* = 8/11). **h** MIX at 18 weeks (ow/ob: *n* = 8/11). Black bars: overweight; white bars: obese. *Statistically significant at alpha < 0.05. ∑All: sum of all BA; ∑Prim: sum of primary BA; ∑Sec: sum of secondary BA; ∑Tert: sum of tertiary BA; 12-α: 12-α hydroxyl BA; non 12-α: non 12-α hydroxylated BA; 12-α/non 12-α: ratio of 12-α hydroxyl BA to non 12-α hydroxylated BA; Conj: conjugated BA; Unconj: unconjugated BA; Conj/Unconj: ratio of conjugated to unconjugated BA

At baseline, BA were comparable between dietary groups (Table 1). Absolute BA in overweight and obese participants combined were not influenced by the respective diets. However, two-way repeated measures ANOVA (weeks 0, 6 and 18; obese vs. overweight; dietary intervention) revealed differences in the summations of the secondary, tertiary, 12-alpha and unconjugated BA over the intervention periods (∑secondary BA: *p* < 0.0001; ∑tertiary BA: *p* = 0.02; ∑12-α: *p* = 0.03; unconjugated BA: *p* = 0.05, respectively). After correction for the baseline, changes in BA between overweight and obese participants were not significant after 6 weeks, but marked and statistically significant at 18 weeks (Fig. 1). This effect was mainly driven by significant increases of BA in obese participants consuming MIX, whereas no significant changes between overweight and obese participants were observed in the control, HCF, and HP-groups (Fig. 2).

Percent protein intake between obese and overweight participants in the respective dietary groups was comparable (obese vs. overweight: control: 21.1 ± 0.7% vs. 20.2 ± 0.8%, *p* = 0.38; HCF: 17.9 ± 0.9 vs. 15.9 ± 0.4%, *p* = 0.08; HP: 26.7 ± 0.4 vs. 26.4 ± 1.4%, *p* = 0.86; MIX: 21.4 ± 1.7 vs. 21.8 ± 0.6%, *p* = 0.64). After 18 weeks, in the entire cohort, higher milk protein intake was the only dietary predictor for raised total BA and primary BA in stepwise regression analyses (*p* = 0.017 and *p* = 0.011, respectively). None of the other protein sources (red meat, fish, poultry, eggs or legumes) showed significant influences (all *p* > 0.18). Milk protein intake explained 7% of changes in total BA and 8% of changes in primary BA.

### Fecal butyrate

Fecal butyrate was not different between the dietary groups, neither at baseline nor throughout the intervention (*p* > 0.57). However, there was a significant difference between overweight and obese participants, both at baseline (*p* = 0.019) and after 18 weeks, when correcting for the baseline (*p* = 0.009). In sub-group analyses, fecal butyrate decreased between 6 and 18 weeks of HP-diet in obese participants only (baseline: 17.2 ± 2.7 mmol/L; week-6: 18.1 ± 2.3 mmol/L, week 18: 11.8 ± 1.5 mmol/L, *p* = 0.048) and did not significantly change in the remaining dietary groups.

### Circulating FGF-19

Fasting FGF-19 plasma levels were not different between dietary groups, neither at baseline nor during the dietary intervention. In the pooled cohort (all 4 dietary groups combined), FGF-19 tended to be higher in obese vs. overweight individuals, both at baseline and throughout the dietary intervention (week-0: 116.6 ± 12.3 vs. 105.54 ± 9.21 pg/mL; week-6: 112.5 ± 11.8 vs. 99.4 ± 8.9 pg/mL; and week-18: 122.6 ± 9.7 vs. 98.0 ± 12.2 pg/mL), but statistical significance was not reached (all *p* > 0.052). When correcting for the baseline, there was a significant effect of the dietary intervention over time for FGF-19 (F_(1.54,103.54)_ = 3.47, *p* = 0.047), but no significant interaction between time and the obese/overweight categorization. A change in fasting FGF-19 plasma levels explained 7% of the changes in total BA in regression analyses (*p* = 0.031).

### Correlation analyses

#### Fecal butyrate correlation analyses

In uncorrected correlation analyses, no correlation between fecal butyrate and breath hydrogen was observed at baseline (*r* = −0.07; *p* = 0.54), but became significant after 6 weeks (*r* = 0.25; *p* = 0.039) and disappeared again after 18 weeks (*r* = 0.16; *p* = 0.22). At baseline, fecal butyrate correlated with both IHL (*r* = 0.26, *p* = 0.046) and *M*-value (*r* = 0.35, *p* = 0.005). Correlations of fecal butyrate with IHL remained significant after 6 weeks (*r* = 0.28, *p* = 0.023) and 18 weeks (*r* = 0.25, *p* = 0.048), but correlations with *M*-value disappeared (6 weeks: *r* = −0.08, *p* = 0.53; 18 weeks: *r* = −0.08, *p* = 0.53). There were no significant correlations of fecal butyrate and BA or FGF-19. After adjusting for age and BMI, no significant correlations of fecal butyrate and above parameters were observed.

### BA partial correlations in the pooled cohort (all 4 dietary groups and overweight and obese participants combined)

Partial correlation coefficients (adjusted for age and BMI) in the pooled cohort between the summations of different BA subsets and IR measures, intrahepatic lipid content (IHL) and FGF-19 are presented in Table 3.

**Table 3:** Partial correlation heat matrix at 0, 6, and 18 weeks between different bile acids (BA) and insulin resistance (IR) measures, intrahepatic lipid content (IHL) and fibroblast growth factor-19 (FGF-19) plasma levels, when corrected for age and body mass index (BMI), in the pooled cohort (*n* = 72; all four dietary groups and overweight and obese participants combined)

	

Bold font denotes significant correlations at *α* = 0.05; strength of color indicates the magnitude of the Pearson’s correlation value (*r*); red indicates positive and blue indicates negative correlations

*∑All* sum of all bile acids (BA) pooled, *∑Prim* sum of primary BA, *∑Sec* sum of secondary BA, *∑Tert* sum of tertiary BA, *12-α* 12-α hydroxyl BA, *non 12-α* non 12-α hydroxylated BA, *12-α/non 12-α* = ratio of 12-α hydroxyl BA to non 12-α hydroxylated BA, *Conj* conjugated BA, *Unconj* unconjugated BA, *Conj/Unconj* ratio of conjugated to unconjugated BA, *HOMA-IR* homeostasis model assessment for insulin resistance, *HEP-IR* hepatic insulin resistance, *FPI* fasting plasma insulin, *M-value* insulin-mediated glucose uptake as a measurement of whole-body insulin sensitivity, *IHL* intrahepatic fat content, *FGF-19* fibroblast growth factor-19

At baseline, only conjugated BA were positively correlated with HOMA-IR (*r* = 0.24, *p* = 0.05) and HEP-IR (*r* = 0.24, *p* = 0.05), but not with FPI and *M*-value.

At week-6, total, primary, tertiarym and conjugated BA correlated significantly with all IR measures. At week-18, most of the observed significant correlations at week-6 remained significant and correlation coefficients were strengthening (Table 3). In addition, correlations of 12-alpha BA and the ratio of conjugated/unconjugated BA with HOMA-IR and FPI became significant. Apart from a positive correlation of IHL with tertiary BA after 6 weeks (IHL: ∑Tert: *r* = 0.26, *p* < 0.001), no relation of IHL with total BA or BA subsets was observed. No significant correlations were noted between FGF-19 plasma levels and IR measures (weeks 0, 6 and 18).

### Partial correlations of BA with IR in the HP and HCF dietary groups (overweight and obese subjects combined)

Significant diet-induced changes of IR were observed in the HP- and HCF groups, but not in control and MIX. Thus, additional correlation analyses were restricted to HP and HCF. In the HP-group, there were no significant correlations between BA and IR measures at baseline, but there were numerous strong and statistically significant correlations of these measures after 6 and 18 weeks of HP-intake (supplementary materials). In the HCF group, no significant correlations were observed, neither at baseline nor after 18 weeks. However, partial correlations of BA with *M*-value were significant for several BA subsets after 6 weeks (supplementary materials).

### Partial correlations between BA and FGF-19

Baseline fasting FGF-19 was positively correlated with most of the BA summations/subsets and negatively correlated with the conjugated/unconjugated BA ratio (Table 3). At week-6, correlations of 12-α, 12-α/non 12-α, and unconjugated BA remained significant (all *p* < 0.05), whereas at week-18 we noted a return to the baseline pattern (Table 3).

**Fig. 3:**
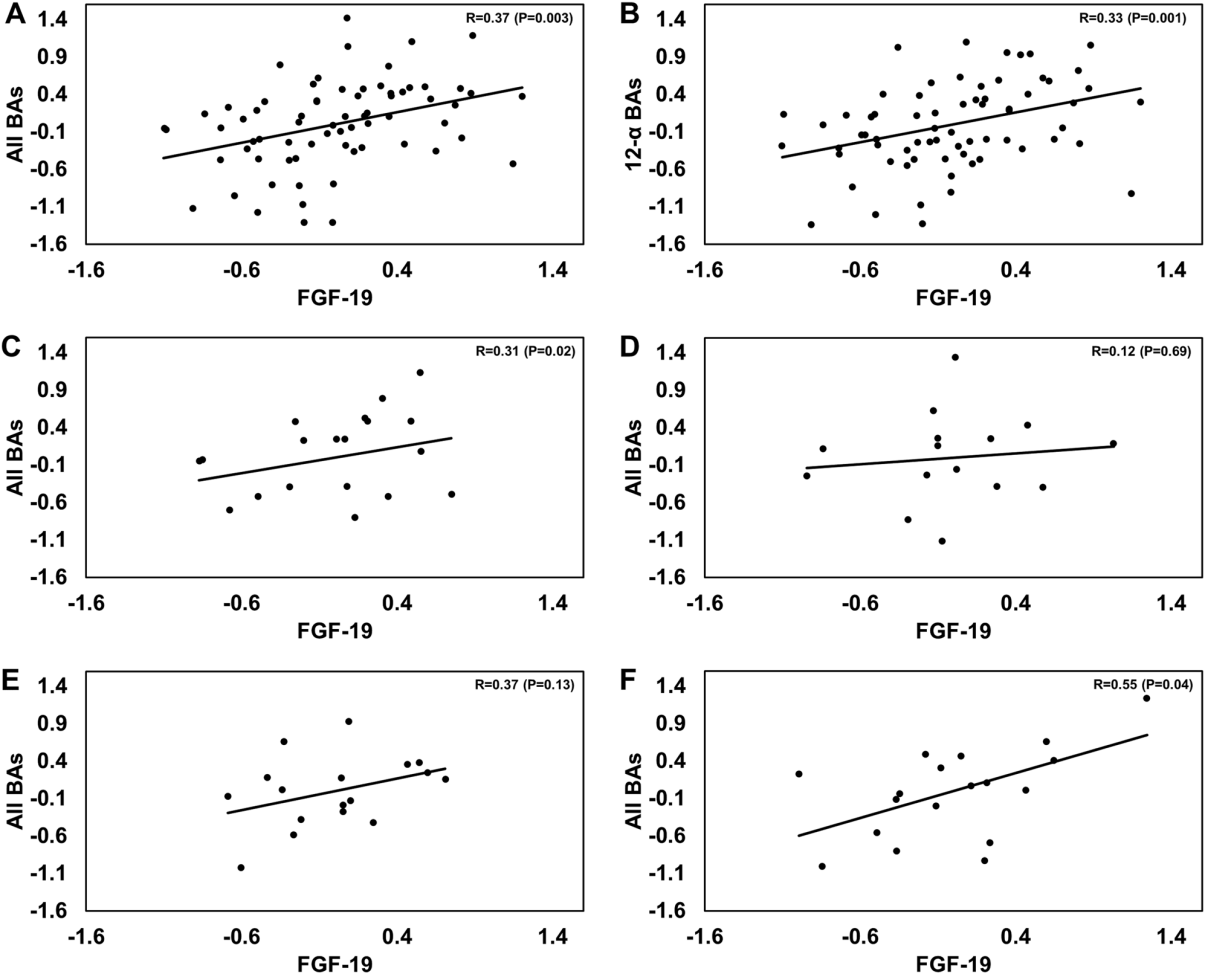
Partial correlation plots for circulating bile acids (BA) vs. fibroblast growth factor-19 (FGF-19) plasma concentrations, corrected for age and body mass index (BMI), measured after 18 weeks of dietary intervention. **a** All study subjects: all BA pooled vs. FGF-19 (*n* = 68). **b** All study subjects: 12-alpha BA vs. FGF-19 (*n* = 68). **c** Control group only: all pooled BA vs. FGF-19 (*n* = 18). **d** High cereal fiber group (HCF) only: all pooled BA vs. FGF-19 (*n* = 15). **e** High protein (HP) group only: all pooled BA vs. FGF-19 (*n* = 17). **f** Mixed-diet (MIX) group only: all pooled BA vs. FGF-19 (*n* = 18). R correlation coefficient

Figure 3 presents the partial correlation plots of FGF-19 with the sum of all BA and 12-α BA at week-18 in the pooled cohort (all four dietary groups combined) (Fig. 3a, b); and partial correlation plots of FGF-19 and the sum of all BAs in each dietary group (Fig. 3c–f). FGF-19 significantly correlated with all BA and 12-α BA in the pooled cohort and in the control and MIX dietary groups, with a similar trend in the HP-diet group. No significant correlation of FGF-19 with BA was observed in the HCF group (Fig. 3d).

## Discussion

Under weight-maintaining conditions, HCF-diets improve and plant based HP-diets worsen IR in subjects at risk of developing T2DM after short-term (6 weeks) exposure, with reversal back to baseline levels after more prolonged (18 weeks) intervention^12^. Herein, we expand on our previous findings by showing (i) significant differences of these diets on IR depending on the degree of adiposity; (ii) effects of these diets on the BA metabolic signatures and their relation to measures of IR; and (iii) investigating the relation of BA signatures and IR with FGF-19, an ileum-derived enterokine which is postprandially induced by BA and has established effects on BA synthesis and glucose metabolism^25, 42, 43^.

Isoenergetic HCF vs. HP intake over 6 weeks changed IR in opposite directions, with significantly improved IR in overweight, but not in obese subjects in the HCF group; and significantly deteriorated IR in obese, but not in overweight subjects in the HP-group. Effects of the prescribed diets on IR weakened after more prolonged (18 weeks) intervention, which could be related to some drop in adherence especially to the HP-diet^12^, or adaptive processes. When adding cereal fibers to HP-intake, worsening of IR in obese participants was prevented and associated with markedly and significantly increased serum concentrations of most BA, whereas BA remained unchanged in overweight, less insulin-resistant participants. Potential mechanisms explaining these effects may include compensatory increases of the BA pool in the insulin-resistant state, including increased synthesis of 12α-hydroxylated BA;^40^ possibly leading to reduced lipogenesis and inhibition of gluconeogenesis in the liver *via* BA induced activation of FXR^15^. Blocking intestinal absorption of BA also increases the flux of BA into the colon and can increase the plasma membrane-bound G protein-coupled receptor TGR5 [TGR5(Gpbar-1)]-mediated release of glucagon like peptide-1, thereby improving IR^15, 44, 45^. However, defective BA transport in the insulin resistant, obese state has been also proposed^40^ and the exact mechanisms leading to different responses of BA depending on both IR and dietary factors remain to be further explored.

The observed interrelations of the BA signature in our study with both specific dietary contents and the degree of adiposity and IR adds to the complexity of the interpretation of recent observations of others, who have shown strong associations of increased circulating BA with IR in both diabetic and non-diabetic populations;^41, 46–48^ and relations between circulating BA and liver fat content^49^, with the latter known to strongly influence IR^50^. In rodents, consumption of a HP-diet increases circulating BA, associated with improvement in metabolic markers^51, 52^. Moreover, in a short-term crossover intervention in 10 healthy normal weight men on a combined high-fat plus HP-diet, circulating BA significantly increased, compared to ingestion of high-fat diet alone^19^, whereas in our study fat consumption was moderately restricted. Further, euglycaemic hyperinsulinaemic clamp studies have shown marked, near 50% suppression of serum BA levels in lean subjects, but impairment of the ability of insulin to decrease BA in obese, insulin-resistant subjects^40^. However, in our study, only HP-induced (but not HCF-induced) changes in IR correlated with significant changes in BA profiles. Further, HP diet-induced worsening of IR in our obese participants only tended to increase circulating BA, whereas effects were striking when adding cereal fiber to the HP-diet (MIX-diet). These findings may indicate that a combination of stimulated BA production by increasing the dietary protein content and modulation of BA absorption via the cereal fiber content may be needed to unmask altered BA metabolic signatures in the obese, insulin-resistant state. Indeed, our finding of major dietary effects on the BA signature with combined HP and cereal fiber intake only is indirectly supported by early observations from Cummings et al.^20^, who had observed, in lean subjects, doubling of BA fecal excretion with combined HP and cereal fiber intake, but no effect when increasing these dietary components isolated. Measurements of fecal BA excretion were not included in our study. However, our finding that plasma BA including secondary BA doubled with combined HCF and HP-intake only (but not when increasing cereal fiber or dietary protein contents isolated) and that this effect was apparent only in obese (more insulin-resistant) participants may suggest increased colonic reabsorption of BA in the obese, IR state, leading to significantly increased plasma BA. Indeed, intestinal permeability is increased in obese, more insulin-resistant patients with hepatic steatosis, compared with obese patients without increased liver fat content^39^, indicating that IR rather than changes in body weight per se could be a key factor in this context. The oat-hull derived cereal fiber supplements used in our studies were neither fermented in vivo, as indicated by absence of changes in hydrogen breath tests over 18 weeks of dietary intervention^12^, nor in vitro^53^. Therefore, significant differences in fecal butyrate concentrations between overweight and obese participants in our study may support altered intestinal permeability in the insulin-resistant state, which could partly explain the observed different diet-induced changes in BA signatures between overweight and obese subjects.

Another finding of the present study was that diet-induced changes in circulating FGF-19 independently associated some of the changes in BA, although this effect was weak. FGF-19 significantly increases in T2DM patients after bariatric surgical procedures, associated with metabolic improvement^54–57^. This appears related to the direct flow of “digestate”-free BA into the ileum after such bariatric operations, which may be more bioactive and induce greater local stimulation of FGF-19^54, 57^. Similarly, it may be hypothesized that when adding cereal fiber to a HP-diet, FGF-19 and BA secretion/levels may be influenced by fiber-related interference of the digestion and/or absorption of dietary protein^12, 58, 59^. Indeed, FGF-19 significantly correlated with total BA in the control and MIX-diet groups, with a similar trend in the HP-group, whereas this correlation was lost in the HCF group. This is also in line with our previous finding of diminished correlations of IR markers with circulating amino acid metabolic signatures in the HCF group only^60^.

Finally, among the various protein sources in this study, only higher milk protein intake was a significant dietary predictor for changes in BA. Of note, non-fat milk has been shown to stimulate FGF-19 expression in vitro, whereas whole-milk had no such effect^57^, possibly related to the higher protein contents in non-fat milk. In our study, all participants had increased their intake of low-fat milk throughout the 18-week intervention, related to the fact that participants in all four dietary groups were instructed to dissolve their twice daily consumed dietary supplements in low-fat milk. This measure may have diminished differences between dietary groups regarding FGF-19 and BA levels.

### Strengths and limitations

Our study has multiple strengths, which include the assessment of the impact of the applied diets on circulating BA profiles in a randomized, controlled intervention with tightly matched groups; use of dietary supplements in all groups to enhance discrimination between diets; strict monitoring of the respective diets including the use of biomarkers of adherence; and use of predefined exclusion criteria for maintaining a stable body weight during the intervention, to avoid potential confounding effects of weight fluctuation on the study outcomes. Furthermore, our protocol employed state-of-the-art methods for the assessment of the principal study outcomes.

However, certain limitations should be acknowledged. The lack of a study group of participants with BMI in the normal range could be viewed as a limitation of the present work, as well as the fact that the number of participants in sub-group analyses was relatively small. However, our protocol allowed us to compare the dietary effects on BA profiles between obese and overweight adults, with the latter constituting a patient population that has been less studied in this context. Further, measurements of fecal BA excretion and the BA precursor serum 7α-hydroxy-4-cholesten-3-one (C4) were not available and we were also not able to explore the potential effects of the applied dietary interventions on postprandial circulating BA profiles, related to the design of our study.

## Conclusion

Our study shows that, under isoenergetic conditions, differences in the protein and cereal fiber contents of a diet can lead to significant changes in both IR and the BA metabolic signatures. These effects appear to be influenced by the degree of IR and adiposity of the respective subjects. Hence, our findings add to existing knowledge and shed light on the complex relationship between BA, IR, and diet manipulations.

## Electronic supplementary material

Supplementary Materials
