# Supplementary material for: Effects of supplemented isoenergetic diets varying in cereal fiber and protein content on the bile acid metabolic signature and relation to insulin resistance
Source: Nutr Diabetes. 2018 Mar 7;8:11. doi: 10.1038/s41387-018-0020-6 (PMC5856807; doi:10.1038/s41387-018-0020-6)
Supplement: Supplementary file 1 — Supplementary Materials [file 41387_2018_20_MOESM1_ESM.docx]

**Supplementary Materials**

**Dietary intervention**

Details of the dietary interventions in the ProFiMet cohort have been published previously (1). In brief, all diets were isoenergetic and based on assumed healthy foods, such as fruit, vegetables, legumes, cereal fiber and dairy products (as listed in **Supplementary Table 1**). The target for fat intake was 30% of the energy content in all four study groups, so that the % fat intake was moderately reduced (30% of energy content) and not significantly different for the four study dietary groups.

All study participants completed food records for three consecutive days (including one weekend day) before the study (baseline) and after 6, 12, and 18 weeks. Moreover, for the initial 6 weeks of the dietary intervention, all study participants weighed all foods whenever possible, and/or provided information on the food brand names, cooking, processing, and household measures. In addition, for the first 6 weeks (42 days) of the dietary intervention, food frequency questionnaires (FFQs) were provided daily, offering a choice of 84 commonly consumed food items suitable for achieving the respective dietary goals. All study participants received feedback at weeks 3 and 6, with adjustment of dietary advice, if appropriate; and all foods noted were coded to foods listed in country-specific food databases. All dietary records were analyzed using the PRODI-4.5-expert software (Nutriscience, Stuttgart, Germany), based on Bundeslebensmittelschluessel (BLS version II3), which includes the nutrient details of approximately 11,400 foods and food preparations (1). The data from the FFQs and the 3-d food protocols were closely correlated.

In the context of this study, the energy requirements for isoenergetic conditions for each study participant were calculated from the subject’s resting energy expenditure (REE) and his/her physical activity level (PAL). Study participants in the four dietary groups received individualized dietary advice in both one-to-one and group sessions in the metabolic unit of the study at weeks 0, 3, and 6, which was further supported by additional telephone recalls at week 1. Moreover, participants were encouraged to freely contact the study nutritionists for additional dietary advice. Based on the information from the food protocols and from the body weight measurements of the study participants, the recommended macronutrient and energy intakes for all study participants were adjusted after 3 weeks (1). Supplementary Table 1 summarizes the typical foods emphasized for the four study groups.

**Dietary supplements**

As previously described in detail (1), in the context of this study, to further support study participants in achieving their respective dietary targets, all participants in the four dietary groups were provided with respective supplements for consumption twice daily over the 18 weeks of the intervention. The supplements were given in four lots (week 0, week 3, week 6, and week 12), were precisely counted, and the exact amount needed was provided to each participant until the next scheduled study visit in the study metabolic unit. Further details on the cereal fiber and protein extracts are listed in the following sections. All supplements for this study were produced in one batch, as coordinated by the Institute for Cereal Processing (Potsdam, Germany), which also performed the analysis of the macro- and micro-nutrient contents of these supplements and sensory tests. Anona Nährmittel Inc (Colditz, Germany) was responsible for the production and packaging of the drinking powders for this study, whilst Kathi Rainer Thiele Inc (Halle, Germany) was responsible for the production and packaging of the baking mixes. All supplements in this study (both for drinking powders and baking mixes) were provided in prepackaged single-portion sachets. In the context of this study, each portion was mixed with 200 mL cold low-fat milk (1.5%) in a purpose-made shaker that was provided to all participants. For the drinking powders produced for the dietary intervention, participants in all groups had a choice between 5 flavors (*i.e.*, banana, caramel, chocolate, vanilla, and white coffee). For preparing the pancakes, 120 mL low-fat milk (fat content: 1.5%; energy and macronutrient contents per 100 mL: 48 kcal, 4.9 g carbohydrates, 3.4 g protein, and 1.6 g fat) was used. All participants were provided with detailed instructions on how to use both drinking powders and baking mixes throughout the intervention, ideally in a 1:1 ratio (1).

*Cereal fiber extracts used for the HCF and MIX dietary interventions*

In the context of this study, a purified fiber extract was used to enrich the provided supplements with cereal fibers in the HCF and MIX groups. This extract was derived from oat hulls, which contained 70% cellulose, 25% hemicelluloses, and 3–5% lignin (Vitacel HF101; Rettenmayr & Soehne Inc, Holzmuhle, Germany), as used in previous studies both in humans and mouse models (2-4). Fiber extracted from oat hulls made up approximately 60% of the total fiber intake in the HCF group and approximately 50% of the total fiber intake in the MIX group. Most of the soluble fiber content, including β-glucans, starch, proteins, and lipids, was removed during the preparation of these products. The details of these processing steps have been previously described (3).

*Protein isolates used for the HP and MIX dietary interventions*

In the context of this study, to enrich the HP and MIX supplements with protein, we used a mixture of 70% whey protein isolate (arla biolac Inc, Harnbarnsen, Germany) and 30% pea protein isolates (Pisane F9; Breuer GmbH, Konigstein, Germany), with an amino acid composition emphasizing leucine and isoleucine and restricting methionine, which was assumed to have beneficial metabolic effects [references provided in (1)].

For the analysis of the proteinogenic amino acid contents in these dietary supplements, samples

were hydrolyzed (6M HCl, 110°C, 18h). For tryptophan analysis, hydrolysis was performed with lithium hydroxide (110°C, 24h). For methionine and cysteine analysis the samples were pre-treated with a mixture of formic acid and hydrogen peroxide (4°C, 16h) before hydrolysis

(6M HCl, 110°C, 18h). After HCL evaporation and washing with water the re-dissolved residues were derivatized with o-phthalaldehyde (OPA) prior to HPLC analysis using fluorescence detection (Agilent). Proline and hydroxyproline were derivatized with 4-chloro-7-nitrobenzofurazan (NBD-Cl). Tryptophan was measured using fluorescence analysis. HPLC was performed using a RP-C18-column and a gradient method (methanol/acetate buffer).

**Bile Acid (BA) measurement**

Elution was performed with a linear gradient from 20% to 98% methanol in water. The organic and aqueous phase contained 0.1% formic acid. The injection volume was 20µl.

In brief, protein precipitation was performed in the presence of stable-isotope labeled internal standards, using a reversed-phase C18 column with 1.8 M particles and a gradient elution at basic pH (5). This allows separation of 18 key BA species (free and conjugated), and a high sensitivity in negative ion mode with detection limits <10 nmol/L. Quantification was achieved by standard addition, and calibration lines were linear in the tested range up to 28 μmol/L. Quantified BA species included cholic acid (CA), chenodeoxycholic acid (CDCA), deoxycholic acid (DCA), lithocholic acid (LCA), ursodeoxycholic acid (UDCA), the glycine (G) conjugated species GCA, GCDCA, GDCA, GLCA, and the taurine (T) conjugated species TCA, TCDCA, TDCA, and TLCA. Validation was performed according to FDA guidelines and the overall imprecision was <11% for all species. The within-assay coefficient of variability (CV) was <8%, while the between-assay CV was <15%. BA standards (labeled and unlabeled) were purchased from Sigma–Aldrich (Taufkirchen, Germany), Steraloids Inc. (Newport, USA), Campro Scientific GmbH (Berlin, Germany), and Larodan Fine Chemicals AB (Malmö, Sweden). All other chemicals used were of analytical grade.

**Measurement of butyric acid in faecal samples**

Fresh faeces (300 mg) were diluted 5 times in water and centrifuged at 15000 x g for 5 min. 23.6 µl 12 mM isobutyric acid (as an internal standard), 280 µl 0.36 M HClO_4_, and 270 µl 1 M NaOH were added to 200 µl of the supernatant. The mixture was lyophilized, and the residue was re-dissolved in 400 µl acetone and 100 µl 5 M formic acid. After centrifugation at 4000 × g for 5 min at room temperature, 1 µl of the supernatant was injected into the gas chromatograph. Authentic standards were incorporated in all runs. Faecal butyrate was measured with an HP 5890 series II gas chromatograph (Hewlett-Packard, Waldbronn, Germany) equipped with a HP-20 M (Carbowax 20 M) column (30 m x 0.53 mm; film thickness 0,3 µm) and a flame ionization detector.

**Partial correlations of BA with IR in the HP and HCF dietary groups (overweight and obese subjects combined)**

In the HP dietary group, at baseline, no significant correlations were observed between BA and any of the IR measures. However, after 6-weeks, we noted strong significant positive correlations of BA with HOMA-IR [∑all BA: r=0.55; ∑tertiary BA: r=0.52, ∑non-12 α BA: r=0.58; ∑non-primary BA (total BA without primary BA): r=0.58; all p <0.05], HEP-IR (∑all BA: r=0.62, ∑non-primary BA: r=0.53; all p <0.05) and FPI (∑all BA: r=0.62, ∑non-12 α BA: r=0.58; ∑non-primary BA: r=0.56; all p <0.05). In addition, non-primary BA negatively correlated with M-value (r= -0.52, p=0.01). At week-18, we observed a re-enforcing of the correlations with HOMA-IR (∑all BA: r=0.60; ∑tertiary BA: r=0.58; all p <0.05), HEP-IR (∑all BA: r=0.82; ∑tertiary BA: r=0.77; ∑non-primary BA: r=0.71; all p <0.05) and FPI (∑all BA: r=0.55; ∑tertiary BA: r=0.50; all p<0.05). Non-primary BA were no-longer correlated with M-value, but tertiary BA (r= -0.61) and conjugated BA (r=0.53) showed strong correlations (all at p<0.05). Also, at week-18, significant correlations to IR measures (correlations which were not present at baseline or week-6) were noted for: 12-alpha BA (HOMA-IR: r=0.57; HEP-IR: r=0.82; FPI: r=0.52, all at p<0.05); unconjugated BA (HOMA-IR: r=0.58; FPI: r=0.52, all at p<0.05); and primary BA (HOMA-IR: r=0.57; HEP-IR: r=0.82; FPI: r=0.52, all at p <0.05).

In the HCF-diet group, no significant correlations were observed at baseline or after 18-weeks of dietary intervention. However, partial correlations of BA with M-value were statistically significant for several BA subsets after 6-weeks (M-value: ∑primary BA: r=-0.58; ∑conjugated BA: r=-0.71; ratio conjugated/unconjugated BA: r= -0.66; all at p<0.05).

**Supplementary Table 1.** Typical example of the corresponding diet characteristics in the 4 intervention study groups [further details are provided in (1)].

| **Diet characteristics ^a^** | **Control ^b^** | **HCF** | **HP** | **MIX** |
| --- | --- | --- | --- | --- |
| Cereal fiber | Not emphasized  < 15 g per 1000 kcal/d | Emphasized  > 20 g per 1000 kcal/d | Not emphasized  < 15 g per 1000 kcal/d | Emphasized  15-20 g per 1000 kcal/d |
| Supplements | Basic supplement [carbohydrates, protein, fat, and cereal fiber: 2 x 28, 8, 3, and 1 g] | Basic supplement enriched with 2 x 15 g cereal fiber extract [carbohydrates, protein, fat, and cereal  fiber: 2 x 25, 8, 3, and 15 g] | Basic supplement enriched with 2 x 29 g protein isolates of whey and peas [carbohydrates, protein, fat, and cereal fiber: 2 x 25, 29,  3, and 1 g] | Basic supplement enriched with 2 x 8 g from cereal fiber and 2 x 19 g protein isolates of whey and peas [carbohydrates, protein, fat, and cereal fiber: 2 x 24, 19, 3, and 8 g] |
| Emphasized foods ^c^ | Bread (refined and whole-meal bread products), fruit, legumes, pasta, potatoes, rice (polished and brown rice products), vegetables | Brown rice, fruit, legumes, potatoes (preferably unpeeled boiled), vegetables, whole-meal bread, whole-meal cereal, whole-meal pasta | Legumes, low-fat dairy products (low-fat milk, cheese, yogurt, low-fat curd cheese), low-fat meat (especially poultry) and fish, low-fat sausages (e.g., ham, chicken breast), vegetables | Fruit, legumes, low-fat dairy products (low-fat milk, cheese, yogurt, curd cheese), pasta, polished rice, potatoes, vegetables, whole-meal bread, wheat and rye bread, whole-meal cereal |
| Moderate intake | Low-fat dairy products (milk, cheese, yogurt, curd cheese), low-fat sausages (e.g., ham, chicken breast), low-fat meat (poultry, fish) | Low-fat dairy products (milk, cheese, yogurt, curd cheese), low-fat sausages (e.g., ham,  chicken breast) | Fruit, pasta, polished rice, potatoes, wheat and rye bread | Low-fat meat (poultry, fish), low-fat sausages (e.g., ham, chicken breast) |
| Restricted | High-fat meat and sausages (e.g., salami, pork sausage, roast pork), jam, honey, spread (butter, margarine), sweets, snacks | High-fat meat and sausages (e.g., salami, pork sausage, roast pork), low-fat protein rich meat (poultry, fish), spread (butter, margarine), sweets, snacks | High-fat meat and sausages (e.g., salami, pork sausage, roast pork), jam, honey, spread (butter, margarine), sweets, snacks | High-fat meat and sausages (e.g., salami, pork sausage, roast pork), jam, honey, spread (butter, margarine), sweets, snacks |
| Emphasized beverages | Water, tea, coffee | Water, tea, coffee | Water, tea, coffee | Water, tea, coffee |
| ^a^ All study participants in the four dietary groups received individualized dietary advice in both one-to-one and group sessions in the metabolic unit of the study at weeks 0, 3, and 6, which was further supported by additional telephone recalls at week 1. For the first 42 days of the dietary intervention food frequency questionnaires (FFQs) were provided daily, offering a choice of 84 commonly consumed food items suitable for achieving the respective dietary goals. All study participants received feedback at weeks 3 and 6. Three-day food protocols were analyzed at weeks 0, 6, 12, and 18. Analyses of food protocols and body weight measurements were used as markers of energy intake with direct analysis and feedback provided to all study participants at weeks 3 and 6. Tailored dietary supplements were used in all the study groups as an additional tool to support participants to achieve their dietary targets. Biomarkers of dietary adherence were used for protein and fermentable fiber intakes.  ^b^ Participants in the control group were instructed to consume a healthy low-fat, moderate-protein, and high-carbohydrate diet, as typically emphasized in the routine nutritional recommendations. In the control group fiber-rich foods were not restricted, but the intake of foods high in insoluble cereal fiber was not particularly emphasized.  ^c^ Listed in alphabetical order.  HCF: diet high in cereal-fiber; HP: diet high in protein; MIX: diet moderately high in both cereal-fiber and protein | | | | |

**Supplementary Table 2**. Categorization of bile acids (BA) into primary (prim), secondary (sec), tertiary (tert), hydroxylated (hyo), 12-alpha (12α) and conjugated (conj) BA.

|  | **Prim** | **Sec** | **Ter** | **Hyo** | **12α** | **Conj** |
| --- | --- | --- | --- | --- | --- | --- |
| TUDCA |  |  | **X** |  |  | **X** |
| GUDCA |  |  | **X** |  |  | **X** |
| UDCA |  |  | **X** |  |  |  |
| GHDCA |  |  |  | **X** |  |  |
| THDCA |  |  |  | **X** |  |  |
| HDCA |  |  |  | **X** |  |  |
| TCA | **X** |  |  |  | **X** | **X** |
| GCA | **X** |  |  |  | **X** | **X** |
| CA | **X** |  |  |  | **X** |  |
| TCDCA | **X** |  |  |  |  | **X** |
| GCDCA | **X** |  |  |  |  | **X** |
| CDCA | **X** |  |  |  |  |  |
| TDCA |  | **X** |  |  | **X** | **X** |
| GDCA |  | **X** |  |  | **X** | **X** |
| DCA |  | **X** |  |  | **X** |  |
| TLCA |  | **X** |  |  |  | **X** |
| GLCA |  | **X** |  |  |  | **X** |
| LCA |  | **X** |  |  |  |  |
| Abbreviations for bile acid species: CA, cholic acid; CDCA, chenodeoxycholic acid; DCA, deoxycholic acid; LCA, lithocholic acid; UDCA, ursodeoxycholic acid; HDCA, hyodeoxycholic acid; G denotes the glycine conjugated species (GCA, GCDCA, GDCA, GLCA, and GHDCA); T denotes the taurine conjugated species (TCA, TCDCA, TDCA, TLCA and THDCA) | | | | | | |

**References Supplementary Materials**

1. Weickert MO, Roden M, Isken F, Hoffmann D, Nowotny P, Osterhoff M, et al.: Effects

of supplemented isoenergetic diets differing in cereal fiber and protein content on insulin

sensitivity in overweight humans. *Am J Clin Nutr* **94*,*** 459-71, 2011.

2. Isken F, Klaus S, Osterhoff M, Pfeiffer AF, Weickert MO: Effects of long-term soluble

vs. insoluble dietary fiber intake on high-fat diet-induced obesity in C57BL/6J mice. *J*

*Nutr Biochem* **21*,*** 278-84, 2010.

3. Weickert MO, Möhlig M, Koebnick C, Holst JJ, Namsolleck P, Ristow M, et al.: Impact

of cereal fibre on glucose-regulating factors. *Diabetologia* **48*,*** 2343-53, 2005.

4. Weickert MO, Möhlig M, Schofl C, Arafat AM, Otto B, Viehoff H, et al.: Cereal fiber

improves whole-body insulin sensitivity in overweight and obese women. *Diabetes Care*

**29*,*** 775-80, 2006

5. Scherer M, Gnewuch C, Schmitz G, Liebisch G: Rapid quantification of bile acids and

their conjugates in serum by liquid chromatography-tandem mass spectrometry. *J*

*Chromatogr B Analyt Technol Biomed Life Sci* **877*,*** 3920-5, 2009.
